# Major Depressive Disorder and Chronic Fatigue Syndrome Show Characteristic Heart Rate Variability Profiles Reflecting Autonomic Dysregulations: Differentiation by Linear Discriminant Analysis

**DOI:** 10.3390/s23115330

**Published:** 2023-06-04

**Authors:** Toshikazu Shinba, Daisuke Kuratsune, Shuntaro Shinba, Yujiro Shinba, Guanghao Sun, Takemi Matsui, Hirohiko Kuratsune

**Affiliations:** 1 Department of Psychiatry, Shizuoka Saiseikai General Hospital, Shizuoka 422-8527, Japan; 2 Research Division, Saiseikai Research Institute of Health Care and Welfare, Tokyo 108-0073, Japan; 3 Autonomic Nervous System Consulting, Shizuoka 420-0839, Japan; 4 Fatigue Science Laboratory Inc., Osaka 532-0011, Japan; 5 Department of Medical Science on Fatigue, Osaka City University Graduate School of Medicine, Osaka 545-8585, Japan; 6 Graduate School of Informatics and Engineering, The University of Electro-Communications, Tokyo 182-8585, Japan; 7 School of System Design, Tokyo Metropolitan University, Tokyo 191-0065, Japan; 8 Department of Metabolism, Endocrinology, and Molecular Medicine, Osaka Metropolitan University Graduate School of Medicine, Osaka 545-0051, Japan; 9 FMCC Co., Ltd., Osaka 532-0011, Japan; 10 Division of Health Science, Osaka University Graduate School of Medicine, Osaka 565-0871, Japan

**Keywords:** heart rate variability, autonomic dysregulation, response to task load, major depressive disorder, chronic fatigue syndrome, linear discriminant analysis, differential diagnosis

## Abstract

Major depressive disorder (MDD) and chronic fatigue syndrome (CFS) have overlapping symptoms, and differentiation is important to administer the proper treatment. The present study aimed to assess the usefulness of heart rate variability (HRV) indices. Frequency-domain HRV indices, including high-frequency (HF) and low-frequency (LF) components, their sum (LF+HF), and their ratio (LF/HF), were measured in a three-behavioral-state paradigm composed of initial rest (Rest), task load (Task), and post-task rest (After) periods to examine autonomic regulation. It was found that HF was low at Rest in both disorders, but was lower in MDD than in CFS. LF and LF+HF at Rest were low only in MDD. Attenuated responses of LF, HF, LF+HF, and LF/HF to task load and an excessive increase in HF at After were found in both disorders. The results indicate that an overall HRV reduction at Rest may support a diagnosis of MDD. HF reduction was found in CFS, but with a lesser severity. Response disturbances of HRV to Task were observed in both disorders, and would suggest the presence of CFS when the baseline HRV has not been reduced. Linear discriminant analysis using HRV indices was able to differentiate MDD from CFS, with a sensitivity and specificity of 91.8% and 100%, respectively. HRV indices in MDD and CFS show both common and different profiles, and can be useful for the differential diagnosis.

## 1. Introduction

In the treatment of patients suffering from fatigue, major depressive disorder (MDD), and chronic fatigue syndrome (CFS), their proper differentiation is important to administer adequate care to patients [1]. Pharmacotherapy, including antidepressants as well as cognitive and behavioral therapy, can be an effective treatment for MDD, whereas the current treatments for CFS are supportive and palliative [2]. An accurate diagnosis will lead to the proper pharmacological treatment and psychosocial support. However, a differential diagnosis is often difficult due to the overlapping psychological and somatic symptoms in both disorders, including fatigability, a lowering of concentration, and sleep disturbances [3,4,5,6]. Depressiveness itself is often present in CFS [7].

MDD is a psychiatric disorder associated with depressed feelings and anhedonia, leading to disturbances in daily life activity. Its diagnosis is based on psychological and behavioral assessment [8,9], and fatigue is listed as one of the symptoms in its diagnostic criteria. Biological alterations in MDD have been reported, including genetic and molecular changes, disturbed brain circulation, and autonomic dysregulation [10], but are not yet included in its diagnostic criteria [8,9].

CFS is a somatic disorder characterized by debilitating fatigue that is not relieved by rest, and is associated with physical signs and symptoms including post-exertional malaise, sleep disturbance, impaired concentration, pain, and tender lymph nodes [11,12]. Biological factors related to its etiology are not yet confirmed, but are assumed to include infectious, immune, inflammatory, molecular, brain-circulatory, and autonomic changes [4,13,14]. Among infectious agents, herpes, influenza, and corona viruses have been reported in relation to CFS [15,16]. It is also argued that the residual symptoms of COVID-19 infection are similar to CFS [17].

Based on these findings on MDD and CFS, biological measures for the differentiation of these two disorders have been investigated. In a study with single-photon emission-computed tomography, a lower frontal blood flow was found in MDD compared with CFS [18]. It has also been suggested that peripheral blood mononuclear cell beta-endorphin concentration can be a diagnostic parameter for differentiation [19]. Skin conductance and temperature showed differences between MDD and CFS [20]. However, these biological measures have yet to be practically used for the differentiation of these disorders. The present study examined the usefulness of heart rate variability (HRV) indices. HRV is a fluctuation of the heartbeat interval and is controlled by the activity of the autonomic nervous system [21]. Various time-domain and frequency-domain parameters of fluctuation are calculated and used to examine autonomic activity [22]. HRV was initially employed to evaluate the severity of cardiac disorders, especially in patients with ischemic heart disease. Then, its use was broadened to various somatic and psychiatric disorders, including MDD and CFS.

Previous research on HRV in MDD and CFS has mostly showed similar abnormalities. At rest, the high-frequency (HF) component is reduced, and the ratio of the low-frequency (LF) component to HF (LF/HF) and heart rate (HR) are elevated in both disorders [6,23,24,25,26,27]. Furthermore, alterations in HRV responsiveness to task load and recovery after a task were found in both disorders [24,25,28,29]. These studies suggest that MDD and CFS are accompanied by a lowered activity and disturbed reactivity of the parasympathetic system, and HRV indices can be used to understand the pathophysiology. However, a detailed comparison of HRV parameters between MDD and CFS has not been performed to examine whether HRV abnormalities can be used for differentiating these disorders in both quantitative and qualitative ways.

The present study used a three-behavioral-state paradigm composed of rest, task, and rest-after-task measurements to analyze the baseline activity, the reactivity to task load, and the recovery after the task in MDD and CFS. Linear discriminant analysis was employed to verify the use of HRV indices in multiple behavioral states for differentiating these two disorders. Our data indicate that the assessment of HRV responses to tasks, as well as that of baseline HRV, are informative. Similarities and differences in HRV measures in MDD and CFS revealed by the present paradigm would lead to the development of an HRV check system for the differential diagnosis of MDD and CFS, enabling appropriate treatment.

## 2. Materials and Methods

### 2.1. Study Design and Participants

In this case–control study, using HRV indices as variables, 3 groups of subjects, the patients with MDD, the patients with CFS, and the healthy controls, were employed. There were 49 patients with MDD (age: 44.0 ± 13.1 years, mean ± s.d.; 20 males and 29 females), 44 patients with CFS (age: 41.8 ± 9.0 years; 11 males and 33 females) and 46 control subjects (age: 40.4 ± 12.1 years; 20 males and 26 females). MDD and CFS were diagnosed using the criteria of the Diagnostic and Statistical Manual of Mental Disorders, 5th edition (DSM5) [8], and with the 1994 Center for Disease Control clinical criteria [11], respectively. Based on these two criteria, subjects with other disorders producing fatigue were excluded. The details of the diagnostic processes can be found in the literature [8,11].

The patients with CFS were recruited consecutively at Osaka City University Hospital. The age- and gender-matched patients with MDD and control subjects were collected at Shizuoka Saiseikai General Hospital. A Kruskal–Wallis test and a chi-square test revealed that the age and male-to-female ratio of the patients with MDD and CFS, and the control subjects, showed no statistically significant differences (age, Ks = 2.795, *p* > 0.05; male-to-female ratio, χ^2^ = 3.860, *p* > 0.05). No subjects had a history of neurological or cardiological disorders, or any other psychiatric disorders. The MDD and CFS patients were naïve to the antidepressant medication.

### 2.2. Ethical Background

The present study was performed in accordance with the Declaration of Helsinki. Written informed consent to participate in this study was obtained from all subjects. The protocol of the study was approved by the Institutional Review Board of Shizuoka Saiseikai General Hospital (No24-10-03).

### 2.3. Heart Rate Variability Measurement

The measurement protocol was the same as that used in our previous studies [24,25]. An adaptation period of at least 5 min was introduced before the start of the experiment. During the experiment, the subject was seated on a chair with the electrocardiogram (ECG) electrodes attached to the chest (RF-ECG2, GM3, Tokyo, Japan). The ECG was measured conventionally, with a gain of 10,000 and a time constant of 0.1 s, and the signals were stored on a computer for offline analysis (Bonaly Light, GMS, Tokyo, Japan). R peaks were used to create the R–R interval trend data, and their fluctuations were analyzed using the maximum entropy method (MemCalc, GMS, Tokyo, Japan). The maximum entropy method was selected for the power spectrum analysis because it has been successfully applied to trend data with a minimum duration of 30 s, and it is useful for studies incorporating measurements of multiple behavioral states [30,31].

In the present system, ECG was recorded and stored in a computer with a sampling frequency of 200 Hz. After peak detection, R–R intervals in the range of 273 and 1500 ms were used for analysis to exclude paroxysmal heart beats. When an R–R interval was omitted, it was replaced by the average of the preceding and following intervals. These R–R intervals were resampled at the mean heart rate.

Using these R–R interval data, MemCalc [32] calculated low-frequency (LF) and high-frequency (HF) components of the spectrum every 2 s by integrating the power at corresponding frequency intervals (0.04–0.15 Hz for LF, 0.15–0.4 Hz for HF) [22] for the preceding 30 s period. Very low-frequency components with a frequency of less than 0.04 Hz were not calculated because the data length for power spectrum analysis was 30 s in the present study and was too short for that component. R–R intervals were also converted to the HR (/min). Previous pharmacological studies have shown that HF reflects parasympathetic activity related to respiratory frequency [21]. Respiration was monitored, and its frequency was confirmed to be within the range of 0.15–0.4 Hz in each subject, as previously indicated [33]. When the respiratory frequency was found to be out of this range, the subjects were informed to modulate their breathing, and then the measurement restarted from the beginning.

The ECG was recorded in three different conditions: rest, task and post-task rest periods (AMAS, GM3, Tokyo, Japan). First, the subjects were instructed to relax as much as possible in the chair for approximately 60 s (the initial rest condition; Rest). Then, the subjects were engaged in a random number generation task for 100 s (the task condition; Task). After the task, ECG was recorded for another 60 s period in the relaxed state (the post-task rest condition; After).

LF, HF, the sum of LF and HF (LF+HF), LF/HF, and HR were averaged in the interval from 30 s after the onset to the end of each condition to exclude any data at the beginning of each new period that may still reflect the previous condition (AMAS, GM3, Tokyo, Japan.

In the random number generation task, the subjects were instructed to orally generate a random series of 100 digits using the numbers 0 through 9 at a rate of 1 Hz. The generation rate was indicated by a metronome click sound. They were requested to concentrate on this task as much as possible. All the subjects completed the task. To evaluate randomness in the generated digit series, counting bias (CB; frequency of counting up or down), interval bias (IB; frequency of same interdigit intervals), and random number generation index (RNG; frequency of same digit pairs) were calculated according to our previous study [34].

### 2.4. Statistics

The differences in the HRV indices at the Rest, Task, and After periods in each subject group were checked with a repeated-measure ANOVA with post hoc Tukey’s multiple comparison test. The differences in the HRV indices in each period among MDD, CFS, and control groups were examined using ANOVA with post hoc Tukey’s multiple comparison test. The differences in the task performance indices (CB, IB and RNG) and in age among MDD, CFS, and control groups were checked using the Kruskal–Wallis test with post hoc Dunn’s multiple comparison test. The male-to-female ratio of the subjects was examined using a chi-square test (Prism 8, GraphPad Software, San Diego, CA, USA).

### 2.5. Linear Discriminant Analysis

In order to further examine the usefulness of HRV indices for differentiating MDD, CFS, and control groups, linear discriminant analysis was employed in the present study. The details can be found in our previous publication [35]. Discriminant analysis was performed to make a linear equation composed of the HRV indices multiplied by coefficients and a constant (Equation (1)). For the HRV indices in the equation, HF, LF, and LF/HF values during the Rest, Task, and After periods, respectively, were used. LF+HF was not included, to avoid the repeated use of LF and HF. LF/HF was employed because the ratio of LF and HF yields new aspects of HRV.

In the three equations, for discriminating MDD from control, CFS from control, and MDD from CFS, the values of the coefficients were set so that the discriminant score (D-score, Equation (1)) could most effectively discriminate between the two groups by making the D-score positive when the analysis supported the former diagnosis, and negative when it supported the latter diagnosis (StatMate V, ATMS, Chiba, Japan).

Sensitivity and specificity in discriminating the former diagnosis from the latter using the D-scores were calculated in the three sets of groups: MDD vs. control, CFS vs. control, and MDD vs. CFS.

Discriminant score (D-score) = a HF[Rest] + b HF[Task/Rest] + c HF[After/Rest]
 + d LF[Rest] + e LF[Task/Rest] + f LF[After/Rest]                                                
+ g LF/HF[Rest] + h LF/HF[Task/Rest] + i LF/HF[After/Rest]                            
− discriminant point                                                                                               (1)

## 3. Results

### 3.1. HRV Indices

Results for LF, HF, LF+HF, LF/HF, and HR in the control subjects and in the patients with MDD and CFS, along with the statistical results, are shown in Table 1 (mean ± s.d.). In Table 1, F values were presented where the effects of the period (Rest, Task, After) and of the group (Control, MDD, CFS) were significant according to ANOVA (*p* < 0.05). Statistical differences found by post hoc tests are also indicated in Table 1.

### 3.2. Task Performance

CB, IB, and RNG scores were 0.134 ± 0.074, 0.583 ± 0.122, and 0.344 ± 0.056 in the control subject, 0.168 ± 0.070, 0.590 ± 0.117, and 0.332 ± 0.035 in MDD, and 0.129 ± 0.087, 0.552 ± 0.125, and 0.311 ± 0.047 in CFS, respectively (mean ± s.d.). The Kruskal–Wallis test indicated that the effect of the group (control, MDD, and CFS) was not significant for CB (Ks = 5.242, *p* > 0.05), for IB (Ks = 1.736, *p* > 0.05), or for RNG (Ks = 5.614, *p* > 0.05).

### 3.3. Linear Discriminant Analysis

**Figure 1:**
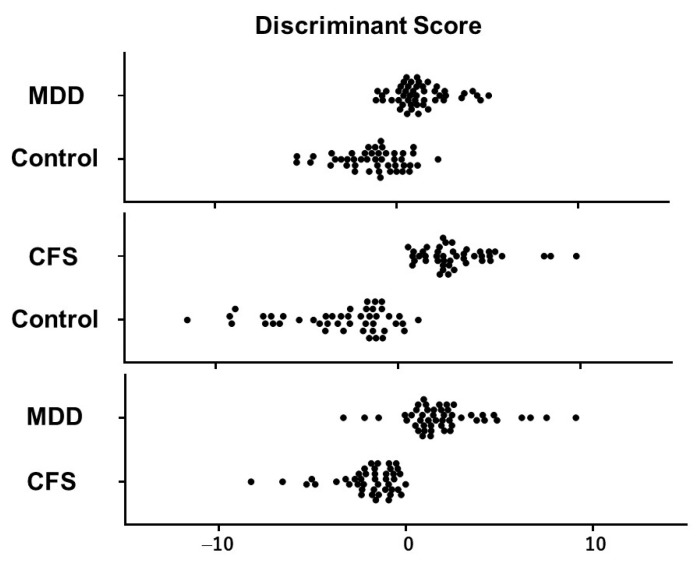
Distribution of discriminant scores (D-scores) in the linear discriminant analyses for MDD vs. Control, CFS vs. Control, and MDD vs. CFS. Each filled circle indicates the individual data.

Three sets of coefficients (a to i) for the HRV indices (LF, HF, and LF/HF) in the discriminant equation (Equation (1)) were determined using linear discriminant analysis to differentiate MDD from the normal control, CFS from the normal control, and MDD from CFS most effectively. The distribution of D-scores in the linear discriminant analysis of MDD and normal subjects, CFS and normal subjects, and MDD and CFS subjects is presented in Figure 1. The number of subjects with positive and negative D-scores, as well as the sensitivity and specificity, are presented in Table 2.

The Mahalanobis distances indicated that discrimination using D-scores is possible, not only between MDD and normal control subjects, and between CFS and normal control subjects, but also between MDD and CFS subjects (Table 2). The discriminant equations with coefficients for LF, HF, and LF/HF during the Rest, Task, and After periods could discriminate between MDD, CFS, and control.

## 4. Discussion

This study examined the HRV indices in MDD and CFS during a paradigm composed of three behavioral conditions: Rest, Task, and After. The HRV scores at these three conditions were incorporated into the analysis, as well as the responsiveness to task load, and the recovery from the response after the task. The results indicated that the HRV indices in MDD and CFS showed differences from those in the control, possibly reflecting the autonomic dysregulations in each disorder. We suggest that the characteristic patterns of HRV profiles in MDD and CFS can be used as physiological biomarkers. HRV is unique as a measurement because it enables the assessment of the task load as well as the baseline condition within a short measurement time, and can reveal different pathophysiological aspects of MDD and CFS in comparison with other methods using imaging and biochemical markers. Future studies are warranted to use multiple physiological markers, such as skin conductance and temperature, in addition to HRV for a thorough understanding of autonomic profiles.

The patients were naïve to anti-depressant medication, and the task performances of the subjects were not different when evaluated with CB, IB, and RNG scores of the random number generation task. It was indicated that the present results were not significantly influenced by the difference in pharmacotherapy or in the behavioral condition during the task.

### 4.1. Normal HRV Pattern

The normal pattern of HRV profiles during the present task, incorporating the three behavioral periods, has already been reported in our previous studies [24,31]. HF decreases, and LF/HF and HR increases during the Task period returned to the baseline level in the After period. These changes in HRV in response to the task load are considered to represent normal autonomic regulation. During the task load with elevated arousal, the balance is shifted toward sympathetic activation. In contrast, when the subject is at rest, parasympathetic activity is dominant. This natural reactivity of the autonomic system in response to arousal changes is important in executing various behaviors.

The functions of LF and HF heartbeat modulations are possibly related to the stabilization of blood flow, which is affected by blood pressure fluctuation and breathing activity, respectively [36]. HR increases when blood pressure is low, and decreases when it is high, producing the LF rhythm of HRV. HR increases with the inspiration of air, and decreases on expiration, creating the HF rhythm. During the Rest period, the power spectrum analysis indicated that the powers of LF and HF are mostly at the same level, resulting in an LF/HF score around 1. In the Task period, both LF and HF tended to decrease, but the reduction is greater for the HF, leading to an increase in LF/HF. This increase in LF/HF has been analyzed as being related to sympathetic activation, although this interpretation has been questioned [37]. After its decrease during the Task period, LF shows an increase during the After period, exceeding its level at Rest. This excessive increase was transient and returned to the baseline level within a few minutes [25]. The increase in LF after the task load could have been a normal autonomic response, although its functional significance has to be assessed in future research.

These HRV and HR profiles in the paradigm with three behavioral periods are informative for understanding the characteristics of autonomic regulation, not only in the healthy condition, but in various disorders including MDD and CFS.

### 4.2. LF Profiles in MDD and CFS

The present study revealed that a low LF at Rest is found in MDD, but not in CFS. It is suggested that the baseline LF reduction is one of the characteristic properties of MDD. On the other hand, the attenuation of the response of LF to the task was observed in both MDD and CFS, and is considered to be common to both disorders.

LF is the heart rate modulation intended to stabilize blood flow in response to Mayer wave blood pressure fluctuation, as described above [36]. LF can help in maintaining a constant blood supply to the tissue by adjusting the heart rate in response to blood pressure changes that occur in various conditions. It is speculated that MDD is deficient of such modulatory activity at rest, and may lead to an unstable blood supply, which may cause both psychological and somatic symptoms. CFS is found to maintain this autonomic function.

At task load, in the control subject, the LF modulation of HR was diminished. This response was weakened in both MDD and CFS, as reflected in the absence of significant change during the task. After the task, LF increased and exceeded the baseline level in MDD, but showed no significant change in CFS. These responses of LF to the task load show large inter-subject differences which need to be further assessed in future research to clarify their functional significance.

### 4.3. HF Profiles in MDD and CFS

The HF data in MDD and CFS indicated that, in both disorders, the score was low at Rest, was not reduced at Task, and was elevated excessively at After. The results showing common disturbances of HF confirmed the previous reports on HRV in MDD and CFS, as described in the introductory section. The HF profile, exhibiting a reduction in baseline parasympathetic activity, together with unresponsiveness to the task load, was qualitatively similar in both disorders.

Furthermore, the results indicated that an excessive increase during the After period, which was not observed in the control, was present in both disorders. The physiological significance of the excessive increase in HF was not clear in this study, and could be different from that in LF because the excessive increase in LF was present in the control. Further study is necessary to assess this issue. However, its presence in both disorders suggests that this phenomenon for HF could reflect the common disturbances of MDD and CFS, including fatigue.

Although these HF profiles were mostly common to both disorders, the present study newly revealed that the magnitude of HF changes is greater in MDD than in CFS. The HF scores at Rest and After were low in MDD and CFS, but the scores in MDD were significantly lower than those in CFS. These observations suggest that the parasympathetic activity in MDD was affected more profoundly than that in CFS. The HF profiles in MDD and CFS were qualitatively similar, but quantitatively different. A severe reduction in the parasympathetic activity may characterize autonomic dysregulation in MDD.

HF represents the control of heart beats and blood flow in response to breathing movements. Both the breathing-related regulation of blood flow at rest and its inhibition during the task load were disturbed in MDD and CFS, which may have led to the generation of the psychological and somatic symptoms of these disorders.

### 4.4. LF+HF Profiles in MDD and CFS

LF+HF is the sum of LF and HF, and fundamentally represents the total HRV in the present paradigm, employing 30 s intervals for power spectrum analysis, which are too short for the calculation of very low-frequency components. The LF+HF scores at Rest, Task, and After in MDD were lower than that in the control and CFS, indicating that total HR modulation is reduced in MDD. On the other hand, the unresponsiveness to task load was common to both disorders. LF+HF is informative in understanding the overall features of autonomic derangement, and in helping to differentiate between these two disorders.

### 4.5. LF/HF Profiles in MDD and CFS

The present study revealed an elevated LF/HF at Rest observed in CFS but not in MDD, in contrast to our previous reports on MDD. However, the increase in LF/HF at Rest in MDD in comparison with the control was statistically significant when the t-test was used (t = 2.994, *p* = 0.004), suggesting that it is common to both MDD and CFS. The physiological significance of high LF/HF is not clear, but should reflect the autonomic condition, where the heart rate modulation for the blood-pressure-related changes in blood flow are greater than those for the breathing-related changes in blood flow. Disturbances in blood pressure control in MDD and CFS may be related to LF/HF data, which warrants future studies.

During the Task period in the control, both LF and HF tended to decrease in comparison with those in the Rest period. However, the reduction was greater for HF, producing an elevation of LF/HF. In MDD and CFS, changes in LF and HF during the Task period were both attenuated, leading to the absence of an LF/HF response.

These changes in LF/HF are common to MDD and CFS, and alone are not capable of differentiating between the two disorders.

### 4.6. HR Profiles in MDD and CFS

The HR profiles were also mostly similar in both disorders. HR was high at Rest and increased during the Task period. In MDD, the HR at After was lower than the HR at Rest, although the functional significance of this change was not clear. High HR has been reported in MDD previously, and was also found in CFS in the present study. Both MDD and CFS are possibly accompanied by an autonomic balance shift toward sympathetic activation, leading to an increase in HR, although the response in the Task period was maintained in both disorders. Overall, HR measurement is important for understanding the existence of autonomic dysregulation, but is not useful for differentiating these two disorders.

### 4.7. Different Profiles of HRV Indices in MDD and CFS

For the differentiation of MDD and CFS, psychological assessment has frequently been employed. Self-reproach, decreased self-esteem, and cognitive distortion are found in MDD, and low ratings of health status, illness identity, and external attributions for their illness are specific to those with CFS [7,38]. However, it has also been argued that persons with CFS should be evaluated for concurrent depression [1].

In CFS, central nervous system inflammation is presumed to be its etiological background, often showing some signs of infection or an onset following viral infection [13]. However, it has been reported that brain inflammation is also present in depression [39]. Inflammatory exploration may not be enough to differentiate between these two disorders. The hypothalamus–pituitary–adrenal system is often discussed among the etiological factors for depression. However, it has also been reported that CFS patients display cortisol hyposecretion in saliva and plasma [40]. In addition to these studies on psychological, inflammatory, and endocrinological factors to differentiate depression and fatigue, the present study indicates the usefulness of autonomic indices.

HRV in MDD is characterized by an overall reduction, including low LF, low HF, and low LF+HF scores. In CFS, on the other hand, only HF is reduced and the reduction is less severe than that in MDD. Total HRV reduction is characteristic for MDD, and can be used for the differentiation of MDD and CFS.

On the other hand, the absence of an HRV response to the task load is common to both disorders, reflected in LF, HF, LF+HF, and LF/HF changes. During the Task period, in contrast to the control subjects showing a decrease in LF, HF, and LF+HF, and an increase in LF/HF, these indices did not change in MDD and CFS. High LF/HF and HR at Rest, as well as an excessive increase in HF in the After stage, were also common to both disorders.

In summary, a total autonomic reduction reflected in low LF, HF, and LF+HF at Rest may support the diagnosis of MDD. HF reduction is found in CFS, but in a lesser severity. Response disturbances of HRV to Task were observed in both disorders, and would suggest the presence of CFS when baseline HRV is not reduced. The present study has indicated that the HRV indices during the three-behavioral-state paradigm are useful in differentiating between MDD and CFS by clarifying these characteristic HRV profiles.

### 4.8. Differentiation of MDD and CFS with Linear Discriminant Analysis Using HRV Indices

The present study further revealed that linear discriminant analysis using HRV indices recorded during the three-behavioral-state paradigm is useful for differentiating not only MDD from the control (sensitivity: 87.8%, specificity: 76.1%) and CFS from the control (sensitivity: 100.0%, specificity: 91.3%), but also MDD from CFS, with the sensitivity and specificity being 91.8% and 100.0%, respectively (Table 2). Differences in the HRV profiles of MDD and CFS were significant, which enabled their differentiation. Despite the similarities, including the parasympathetic underactivity reported previously, some autonomic activities were distinct in MDD and CFS. Future studies are warranted to use linear discriminant analysis practically for making differential diagnoses of these disorders.

### 4.9. Co-Occurrence of MDD and CFS

The similarities in some aspects of the HRV profiles in the present three-behavioral-state paradigm may have indicated the co-occurrence of MDD and CFS. Fatiguability is one of the main symptoms of MDD [DSM-5], and depressiveness often accompanies CFS [7]. Both disorders may have common pathophysiological backgrounds, reflected in common HRV abnormalities. The response of HRV to the task load was attenuated, suggesting a difficulty in shifting from rest to activation in both MDD and CFS. This shift disturbance may be mentally expressed as depressiveness in MDD and physically expressed as fatigue in CFS. Future studies using other biological parameters will be necessary to assess this issue.

### 4.10. Limitations

There are limitations in the present study. The biographical and clinical profiles of the patients were not fully analyzed in either MDD or CFS. Previous studies have suggested that HRV is related to symptom severity and cognitive disturbance in CFS [27,41]. It would be interesting to examine the effects of the differences in clinical data, including symptom severity, the duration of illness, and laboratory tests in the HRV profiles of both disorders in future studies.

As for the linear discriminant analysis, the equations were based on the present sets of data, and should be tested for new sample groups in future study to verify and improve their effectiveness. Furthermore, newer methods, including support vector machines, have been used for machine learning in medical research and will have interesting applications in future study [42].

It is also important to assess the validity of the present findings by analyzing the data of disorders other than MDD and CFS, including bipolar disorder and stress disorder.

It has already been reported that the HRV changes in MDD are ameliorated by adequate treatment [43], and are state-dependent. In future studies, the longitudinal assessment of patients should be used to clarify whether HRV changes in CFS are also state-dependent [44].

## 5. Conclusions (Highlights)

- HRV indices during the three-behavioral-state paradigm in MDD and CFS used in the present study showed both common and different profiles and could be useful for differential diagnosis.
- The common profiles confirmed previous findings.
- The overall HRV reduction at Rest may support a diagnosis of MDD.
- HF reduction at Rest was found in CFS, but with a lesser severity.
- Response disturbances of HRV to the task load were observed in both disorders, and suggest the presence of CFS when the baseline HRV is not reduced.
- Linear discriminant analysis using HRV indices was able to differentiate MDD from CFS, with sensitivity and specificity being 91.8% and 100%, respectively.

## 6. Patents

The contents of the present study are included in the patents Japanese Patent JP 5,492,247,B2; European Patent EP 2,862,509,B1; and U.S. Patent US 8,852,116,B2.

## Figures and Tables

**Table 1:** Heart rate variability indices (LF, HF, LF+HF, LF/HF) and heart rate (HR) at Rest, Task, and After periods in the normal (Control), major depressive disorder (MDD), and chronic fatigue syndrome (CFS) subjects (mean ± s.d.).

LF	Rest (ms^2^)			Task (ms^2^)			After (ms^2^)				F
Control	780 ± 742			517 ± 475	↓		966 ± 799	↑			10.9
MDD	266 ± 320	▼		340 ± 463			572 ± 821	↑			10.2
CFS	601 ± 979			469 ± 438			802 ± 1328				
F	6.2										
											
HF											
Control	388 ± 368			136 ± 122	↓		438 ± 392				28.6
MDD	77 ± 105	▼		86 ± 127			165 ± 215	↑	▼		15.0
CFS	214 ± 255	▼	#	152 ± 165			395 ± 587	↑		#	9.5
F	16.6						5.8				
											
LF+HF											
Control	780 ± 742			517 ± 475	↓		966 ± 799				10.9
MDD	266 ± 320	▼		340 ± 463			572 ± 821	↑			10.2
CFS	815 ± 1129		#	622 ± 588		#	1196 ± 1656			#	5.4
F	7.2			3.7			3.6				
											
LF/HF											
Control	1.58 ± 1.49			4.10 ± 4.11	↑		1.71 ± 1.37				17.4
MDD	3.34 ± 3.71			4.93 ± 5.00			3.60 ± 3.73		▲		3.2
CFS	4.98 ± 8.99	▲		4.63 ± 3.98			3.19 ± 3.17		▲		
F	4.2						5.3				
											
HR											
Control	73.2 ± 8.51			82.2 ± 9.6	↑		72.4 ± 8.7				58.9
MDD	80.6 ± 12.2	▲		84.4 ± 13.7	↑		79.0 ± 11.9	↓	▲		36.6
CFS	79.2 ± 10.8	▲		85.1 ± 13.1	↑		77.6 ± 12.1				36.0
F	6.4						4.6				

ANOVA F values are presented on the right side of the After column when the effect of the period is significant. Upward (↑) and downward (↓) arrows indicate a significant increase and a decrease from the Rest score, respectively (*p* < 0.05). ANOVA F values are presented at the bottom of the column when the effect of the group is significant. Upward (▲) and downward (▼) triangles indicate that the scores are significantly higher and lower than the Control score, respectively (*p* < 0.05). A number sign (#) indicates the presence of a significant difference between the scores in MDD and CFS (*p* < 0.05). Detailed descriptions of statistics are found in the text.

**Table 2:** Number of Subjects Showing Positive Discriminant Scores (D > 0) and Negative Discriminant Scores (D < 0) in Linear Discriminant Analysis.

	D > 0	D < 0	Total	Mahalanobis d	*p*
MDD vs. Control				2.62961	<0.001
MDD	43	6	49		
Control	11	35	46		
Total	54	41			
					
	sensitivity	specificity			
	87.8%	76.1%			
CFS vs. Control				6.48905	<0.001
CFS	44	0	44		
Control	4	42	46		
Total	48	42			
					
	sensitivity	specificity			
	100%	91.3%			
MDD vs. CFS				4.05344	<0.001
MDD	45	4	49		
CFS	0	44	44		
Total	45	48			
					
	sensitivity	specificity			
	91.8%	100%			

MDD: major depressive disorder, CFS: chronic fatigue syndrome, D: discriminant score in linear discriminant analysis, Mahalanobis d: Mahalanobis distance, P: *p* value for Mahalanobis distance.

## Data Availability

The data that support the findings of this study are available on request from the corresponding author. The data are not publicly available due to privacy and ethical restrictions.
